# Transforming Gerontology: The Power of Minority Aging Research

**DOI:** 10.1093/geroni/igaf122.1525

**Published:** 2025-12-31

**Authors:** Elizabeth Muñoz, Tyrone Hamler

## Abstract

As the field of gerontology continues to evolve, minority aging research remains essential in shaping its future by promoting health equity and advancing inclusive aging scholarship. This session convenes esteemed scholars—past recipients of the James Jackson Outstanding Mentorship Award—who have made significant contributions to minority aging research. Dr. Jacqueline Angel, Dr. Maria Aranda, Dr. Tamara Baker, Dr. Lisa Barnes, and Dr. Roland Thorpe will share insights from their careers, reflecting on the challenges, breakthroughs, and impact of their work. Through a moderated discussion, panelists will provide key advice for emerging scholars seeking to advance research in this area, offering perspectives on navigating the field, fostering mentorship, and driving meaningful change. Attendees will gain a deeper understanding of the complexities and opportunities within minority aging research, learning from the experiences and lessons of leading experts. By drawing from the collective wisdom of these distinguished scholars, this session aims to inspire, support, and equip the next generation of gerontologists to advance equity in aging research and practice.

